# Remarkable Facts Relating to Insanity in the United States

**Published:** 1884-01

**Authors:** 


### REMARKABLE FACTS RELATING TO INSANITY IN THE UNITED STATES.

A valuable paper was recently read on this subject before the American Public Health Association, by Foster

Pratt, M.D., of Michigan, from which we glean the following facts:

First—That the proportion of insane to native white population in the Northern States is 1 in 597; in the Southern States, 1 in 660.

Second—That among the foreign white the proportion in the Northern States is 1 in 248, and in the Southern States, 1 in 283.

Third—That among the colored race the proportion in the Northern States is 1 in 540, and in the Southern States, 1 in 1,235.

These statistics, gleaned from the United States census of 1880, speak volumes of valuable truths, as regards certain social conditions, North and South. The disparity in the case of the negro, North and South, is especially noticeable. The truth of nature, as Thomas Carlyle predicted it would, is gradually asserting itself. But this is only one out of many instances that any intelligent Southerner could designate. In regard to consumption, previous to the late war it was the rarest of all events in the South to find or hear of a genuine darkey dying of phthisis. Now the race is fast making good its right to suffer and die of consumption, as well as to be as free as the white boss. If honest, unprejudiced men would come South and investigate the present condition of the negro, it would not be long before the civilized world would be in possession of some startling facts as the grand results of Wm. Loyd Garrison's abolition crusade, culminating in the famous emancipation proclamation of Lincoln. Thus we progress; but somebody has to pay the fiddler.

